# IL-18 binding protein suppresses IL-17-induced osteoclastogenesis and rectifies type 17 helper T cell / regulatory T cell imbalance in rheumatoid arthritis

**DOI:** 10.1186/s12967-021-03071-2

**Published:** 2021-09-16

**Authors:** Hong Ki Min, Sehee Kim, Ji-Yeon Lee, Kyoung-Woon Kim, Sang-Heon Lee, Hae-Rim Kim

**Affiliations:** 1 Division of Rheumatology, Department of Internal Medicine, Konkuk University Medical Center, Seoul, 05030 Republic of Korea; 2 The Rheumatism Research Center, Research Institute of Medical Science, Konkuk University School of Medicine, Seoul, 05030 Republic of Korea; 3 R&D Center, OncoInsight, Seoul, 06373 Republic of Korea; 4 Division of Rheumatology, Department of Internal Medicine, Research Institute of Medical Science, Konkuk University School of Medicine, Seoul, 05030 Republic of Korea; 5 Department of Rheumatology, Konkuk University Medical Center, 120-1 Neungdong-ro (Hwayang-dong), Gwangjin-gu, Seoul, 143-729 Republic of Korea

**Keywords:** Interleukin-18 binding protein, Rheumatoid arthritis, Osteoclastogenesis, Type 17 helper T cell, Regulatory T cell

## Abstract

**Background:**

Patients with rheumatoid arthritis (RA) have increased levels of interleukin-18 (IL-18) and decreased levels of IL-18 binding protein (IL-18BP) in the serum and synovial fluid (SF) compared to those in patients with osteoarthritis (OA) or in healthy controls. In this study, we evaluated the effects of IL-18BP on osteoclastogenesis and T cell differentiation in RA in vitro.

**Methods:**

Serum and SF of patients with RA and OA were collected to compare IL-18 and IL-18BP levels by the enzyme-linked immunosorbent assay. Peripheral blood mononuclear cells (PBMCs) and SF mononuclear cells (SFMCs) of RA patients were cultured under type 17 helper T cell (Th17) polarisation conditions with or without IL-18BP. In addition, PBMCs were cultured in the presence of receptor activator of nuclear factor kappa-Β ligand (RANKL) or IL-17A with or without IL-18BP, and tartrate-resistant acid phosphatase (TRAP) staining and real-time quantitative polymerase chain reaction for expression levels of osteoclast-related genes were performed.

**Results:**

IL-18 levels were higher in the serum and SF of patients with RA, whereas IL-18BP was lower in the SF of patients with RA than in the control group. Treatment of patients’ PBMCs with IL-18BP decreased the differentiation of CD4^+^ IL-17A^+^ and CD4^+^ RANKL^+^ T cells, whereas the differentiation of CD4^+^CD25^high^FOXP3^+^ T cell population increased in a dose-dependent manner. These changes in CD4^+^ T cell differentiation were also observed in the SFMCs of patients with RA. The levels IL-17A and soluble RANKL in the culture medium were significantly decreased by IL-18BP. IL-18BP administration decreased TRAP^+^ cell counts in a dose-dependent manner on the background of stimulation with RANKL-and IL-17A. In addition, expression levels of *TRAP, NFATC1, CTSK*, and *TNFRSF11A* (*RANK*) genes were lower in the IL-18BP treated cells.

**Conclusion:**

We showed that IL-18BP can rectify the Th17/Treg imbalance and decrease IL-17-induced osteoclastogenesis in PBMCs from patients with RA. Therefore, IL-18BP may have therapeutic potential for RA treatment.

**Supplementary Information:**

The online version contains supplementary material available at 10.1186/s12967-021-03071-2.

## Background

Rheumatoid arthritis (RA) is a chronic inflammatory arthritis that affects approximately 0.5–1% of the general population, and chronic inflammation associated with RA induces irreversible joint destruction, especially in small joints [1]. Although the pathogenesis of RA is complex and dependent on the interactions of various immune cells, osteoclasts, chondrocytes, and synovial lining cells [1], the main pathological immune cause of RA is thought to be a dysregulated adaptive immune reaction. Among the adaptive immune system cells, cluster of differentiation (CD)4^+^ T cells are one of the critical pathological cells in RA pathogenesis [2]. In RA synovium, the CD4^+^ T cell population producing interleukin (IL)-17 is increased [3]. Specifically, hyperactivation of type 17 helper T (Th17) cells and suppression of regulatory T (Treg) cells are the cornerstone pathological hallmarks of RA [2]. In addition, circulating Th17 population and IL-17 levels were found to correlate with disease activity of RA [4], whereas Treg number inversely correlated with the disease activity score [5]. Similarly, in a mouse model of RA, the Th17/Treg imbalance was associated with arthritis progression [2], which implies that Th17/Treg imbalance could be a marker of RA progression.

The irreversible destruction of joints is the hallmark of RA. Hypertrophy of the synovium, also defined as pannus, causes inflammation of the joints and consequently destroys the adjacent cartilage and bone. The osteoclasts act as the main effector cells in joint destruction [1]. Osteoclast precursor cells originate from CD14^+^ monocytes, and interactions between RANK and RANKL mediate osteoclast differentiation and maturation [1, 6]. In inflammatory arthritis, osteoclast differentiation is up-regulated when compared with that in healthy controls; this is due to an increase in inflammation. Several pro-inflammatory cytokines related to RA pathogenesis are known to increase osteoclast differentiation [6, 7]. Therefore, regulating osteoclastogenesis is one of the primary objectives for RA treatment.

IL-18, originally called interferon-γ-inducing factor, belongs to the IL-1 superfamily [8]. IL-18 levels in the RA synovium are higher than in the osteoarthritis (OA) synovium, and IL-18 aggravates arthritis severity in a model of collagen-induced arthritis [8]. Furthermore, IL-18 stimulates Th17 differentiation and IL-17 production, promoting autoimmune diseases [9, 10]. IL-17, one of the main Th17 cytokines, increases the expression of receptor activator of nuclear factor kappa-Β ligand (RANKL) in RA-derived fibroblast-like synoviocytes and can even induce osteoclastogenesis without RANKL stimulation in vitro [11]. IL-18 binding protein (IL-18BP) inhibits the effects of IL-18 [12]. IL-18BP was discovered in 1999 and shown to inhibit Th1 responses and interferon-gamma production [12]. IL-18BP is different from transmembrane IL-18 receptors, in that, it has only one IgG domain [9]. IL-18BP is a secretory protein and acts as a decoy receptor with high affinity for IL-18 (400 pM) [9]. In the collagen-induced arthritis model, overexpression or administration of IL-18BP reduced the severity of arthritis [13, 14]. In the serum and synovial fluid of patients with RA, IL-18 level was increased, whereas IL-18BP level was decreased compared to those in patients with OA or in healthy controls [15, 16]. However, the levels of IL-18 and IL-18BP did not correlate with disease activity (swollen joint count and serum level of C-reactive protein) or structural damage of the joint [17], and IL-18 tends to decrease after six months of methotrexate treatment; however, the differences of IL-18 levels between baseline and post-methotrexate treatment were non-significant [17]. IL-18BP reduced osteoclast differentiation in osteoporotic mice [18], and osteoclasts are the main effector cells of joint destruction in RA [1]. Although these findings suggest that IL-18BP might be used for treatment of RA, the mechanism by which IL-18BP regulates RA pathogenesis has not yet been elucidated.

We aimed to determine IL-18 and IL-18BP expression levels in patients with RA and examine the regulatory role of IL-18BP in Th cell differentiation. Furthermore, the action of IL-18BP on osteoclastogenesis, especially IL-17-mediated osteoclast differentiation, was assessed.

## Methods

### Patients

Serum samples were obtained from 40 patients with RA and 20 patients with OA. The SF samples from 24 patients with RA and 20 patients with OA were also collected. Inclusion criteria for patients with RA were as follows: (1) fulfilment of the 1987 revised criteria of the American College of Rheumatology, 1987 (formerly the American Rheumatism Association) or 2010 classification criteria for RA, and (2) age over 18 years old. Patients with RA were divided into those with active (N = 24) and inactive (N = 16) RA according to disease activity score-28 (DAS28), in which DAS28 ≥ 3.2, was judged as active state. The present study was conducted in accordance with the Declaration of Helsinki and Good Clinical Practice guidelines. Informed consent was obtained from all participants before enrollment. The experimental protocol was approved by the Konkuk University School of Medicine Human Research Ethics Committee (KUH1010186).

### Enzyme-linked immunosorbent assay (ELISA) of IL-18 and IL-18BP levels in serum and synovial fluid

In brief, a 96-well plate (Eppendorf, Hamburg, Germany) was coated with monoclonal antibodies against IL-18, IL-18BP, IL-17A, and soluble RANKL (R&D Systems, Minneapolis, MN, USA) at a concentration of 4 μg/mL at 4 °C overnight. After blocking with phosphate-buffered saline/1% bovine serum albumin /0.05% Tween 20 for 2 h at room temperature (22–25 °C), the test samples and the standard recombinant IL-18, IL-18BP, IL-17A, or soluble RANKL (R&D Systems) were added to the 96-well plate and incubated at room temperature for another 2 h. The plates were washed four times with phosphate-buffered saline/Tween 20, and then incubated with 500 ng/mL biotinylated mouse monoclonal antibodies against IL-18, IL-18BP, IL-17A, or soluble RANKL (R&D Systems) for 2 h at room temperature. After washing, the streptavidin–alkaline phosphate–horseradish peroxidase conjugate (Sigma, St Louis, MA, USA) was added to the wells for 2 h, followed by another wash, and incubation with 1 mg/mL *p*-nitrophenyl phosphate (Sigma) dissolved in diethanolamine (Sigma) to develop the colour reaction. The reaction was stopped by the addition of 1 M NaOH, and the optical density of each well was measured at 405 nm. The lower limit of IL-18, IL-18BP, IL-17A, and soluble RANKL was 10 pg/mL. Recombinant human IL-18, IL-18BP, IL-17A, or soluble RANKL diluted in the culture medium were used as calibration standards, ranging from 10 to 2000 pg/mL. A standard curve was drawn by plotting the optical density against the log of the concentration of recombinant cytokines, and the curve was used to determine IL-18, IL-18BP, IL-17A, or soluble RANKL concentrations in the test samples.

### In vitro* culture of PBMCs under Th17 polarizing condition with IL-18BP*

Peripheral blood mononuclear cells (PBMCs) were extracted from heparinised peripheral blood of RA patients (N = 5) using the standard Ficoll-Paque density gradient method (GE Healthcare Biosciences, Uppsala, Sweden). In addition, synovial fluid mononuclear cells (SFMCs) from patients with RA (N = 20) were stored in a liquid nitrogen tank at  − 200 °C. Cells were cultured in the RPMI-1640 medium (Gibco BRL, Carlsbad, CA, USA) containing penicillin (100 U/mL), streptomycin (100 μg/mL), and 10% foetal bovine serum (Gibco BRL) that had been inactivated by heating to 55 °C for 30 min. Cell suspensions were dispensed into 48-well plates (Eppendorf). The cell culture plate was pre-incubated with an anti-CD3 antibody (1 μg/mL) for 1 h at 37 °C. PBMCs or SFMCs (1 × 10^6^) were plated in 48-well plates (Nunc) with anti-IFN-γ (2 μg/mL) and anti-IL-4 (2 μg/mL) antibodies for 4 h. Then, an anti-CD28 antibody (1 μg/mL), IL-1β (20 ng/mL), IL-6 (20 ng/mL), and IL-23 (20 ng/mL; R&D Systems) were added and cultured for 72 h [19]. To investigate the impact of IL-18BP (R&D Systems) on the regulation of CD4^+^ T cell differentiation, IL-18BP was added at various concentrations (10, 50, and 100 ng/mL).

### Flow cytometry

To quantify IL-17A^+^, RANKL^+^, and CD25^+^ forkhead box P3 (FOXP3)^+^ cells in CD4^+^ T cells, PBMCs cultured under Th17 polarisation conditions with various doses of IL-18BP were immunostained using a PerCP-conjugated anti-CD4 antibody (BD Biosciences, San Jose, CA, USA), then fixed and permeabilised using a Cytofix/Cytoperm Plus kit (BD Biosciences). Following the manufacturer’s instructions, PBMCs were stained with phycoerythrin-conjugated anti-IL-17A (eBiosciences, San Diego, CA, USA), or phycoerythrin-conjugated anti-RANKL (eBiosciences), or allophycocyanin-conjugated anti-CD25 (BD Biosciences) with phycoerythrin-conjugated anti-FOXP3 (BioLegend, San Diego, CA, USA) antibodies. All cells were detected using a FACSCalibur flow cytometer (BD Pharmingen, Franklin Lakes, NJ, USA).

### Osteoclast formation

CD14^+^ monocytes were prepared from PBMCs using microbeads (Miltenyi Biotec, Auburn, CA, USA). Human CD14^+^ monocytes were seeded in 48-well plates at 5 × 10^4^ cells/well in 1 mL of medium. Monocytes were cultured in α-minimum essential medium supplemented with 10% heat-inactivated foetal bovine serum and 25 ng/mL recombinant human macrophage colony-stimulating factor (rhM-CSF, R&D system) for 3 days. Monocytes were then cultured under RANKL (30 ng/mL) + M-CSF (25 ng/mL) or IL-17A (50 ng/mL) + M-CSF (25 ng/mL) for 10 to 14 days. IL-18BP (0, 10, 50, or 100 ng/mL) was also added three days after rhM-CSF stimulation to evaluate its effect on osteoclastogenesis. After 10–14 days of RANKL + M-CSF + IL-18BP or IL-17A + M-CSF + IL-18BP stimulation, tartrate-resistant acid phosphatase (TRAP)-positive cells were identified using a TRAP staining kit (Cosmo Bio, Tokyo, Japan), as described previously [20, 21].

### RNA preparation and quantification of gene expression levels using real-time quantitative polymerase chain reaction

Total RNA was extracted using an easy-spin™ Total RNA Extraction Kit (Intron Biotechnology, Seongnam, Republic of Korea) according to the manufacturer’s instructions. RNA samples were then quantified, aliquoted, and stored at − 80 °C until analysis. Total RNA (500 ng) was reverse transcribed into cDNA using an AccuPower CycleScript RT PreMix cDNA synthesis kit (Bioneer, Daejeon, Republic of Korea) according to the manufacturer’s instructions. RT-qPCR was conducted in a total volume of 20 μL containing 7.2 μL of PCR-grade distilled water, 0.4 μL of forward and reverse primers, and 10 μL of the SYBR Green I Master mix (Roche Diagnostics, Mannheim, Germany). PCR conditions were as follows: 95 °C for 10 min, followed by 35 cycles of 95 °C for 10 s, 55 °C for 10 s, and 72 °C for 10 s. All primers were synthesised by Bioneer Corp. (Daejeon, Republic of South Korea). The relative mRNA expression levels were normalised to β-actin mRNA levels.

### Statistical analysis

All data are expressed as the mean ± standard error of the mean. Statistical analysis was performed using one-way analysis of variance followed by the post hoc Bonferroni’s multiple comparison test. Spearman’s correlation test was used to determine the correlation between serum levels of IL-18 and IL-18BP. Statistical significance was set at *P* < 0.05. All statistical analyses were performed using Prism 9.0 (GraphPad Software Inc., San Diego, CA, USA).

## Results

### Serum and synovial fluid levels of IL-18 and IL-18BP in patients with RA and OA

**Fig. 1:**
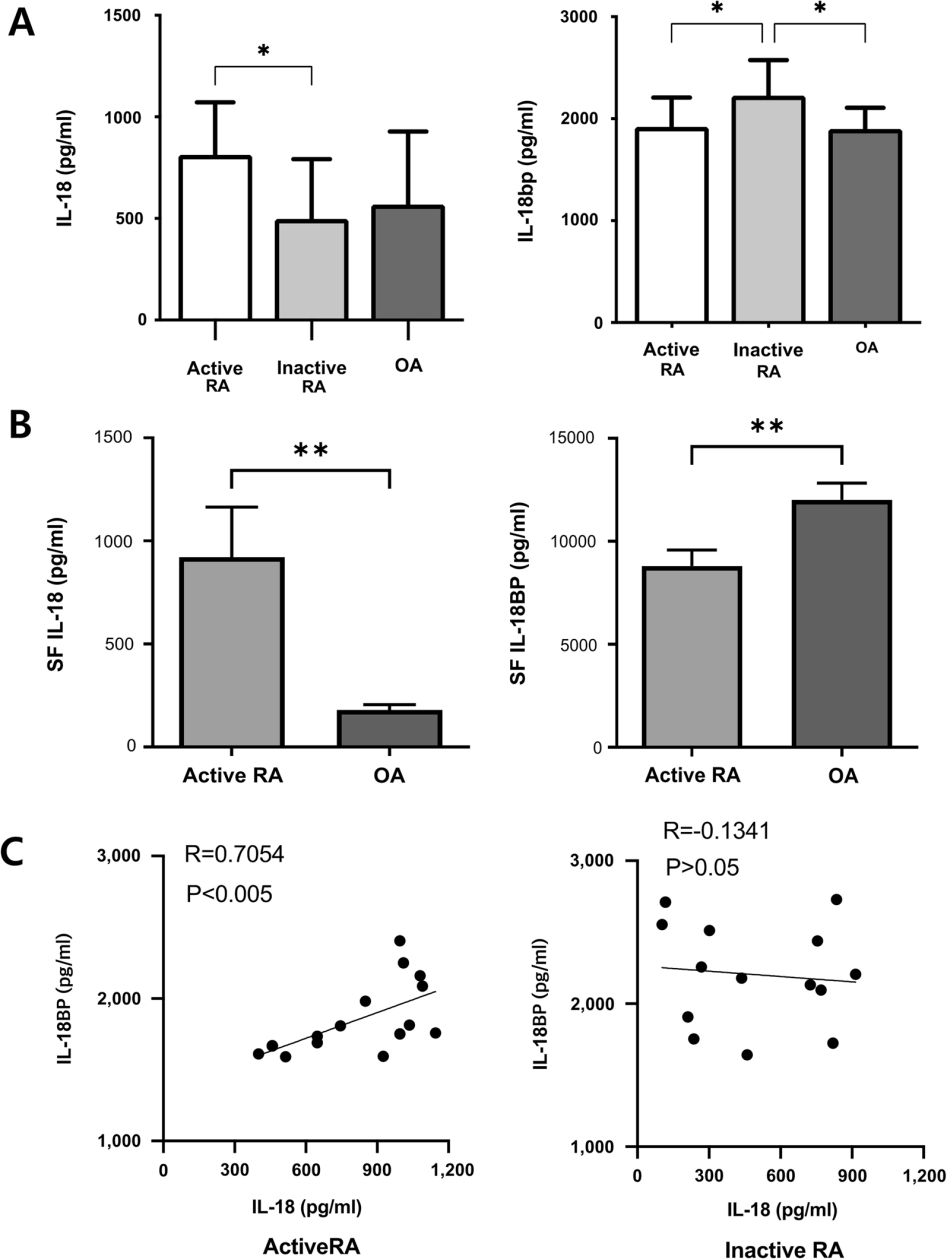
Serum and synovial fluid levels of IL-18 and IL-18BP in patients with rheumatoid arthritis (RA). **A** Serum levels of IL-18 and IL-18BP was measured by ELISA in active, inactive RA and OA patients. **B** IL-18 and IL-18BP levels was also measured in synovial fluid obtained from active RA and OA patients. **C** Correlations between the serum levels of IL-18 and IL-18BP in active and inactive RA. ^*^P < 0.05, ^**^P < 0.001, ^***^P < 0.001

The serum level of IL-18 was higher in patients with active RA (DAS28 ≥ 3.2) than in patients with inactive RA (Fig. 1a). However, the serum level of IL-18BP was lower in patients with active RA than in those with inactive RA (Fig. 1a). Higher IL-18 and lower IL-18BP levels were observed in the SF of patients with active RA than in the SF of patients with OA (Fig. 1b). The serum levels of IL-18 and IL-18BP significantly correlated in the active RA subgroup (*Rho* = 0.705, *P* < 0.05), whereas no correlation was observed in the inactive RA subgroup (Fig. 1c).

### *Modulatory effects of IL-18BP on CD4*^+^*T cells cultured under Th17 polarizing conditions*

**Fig. 2:**
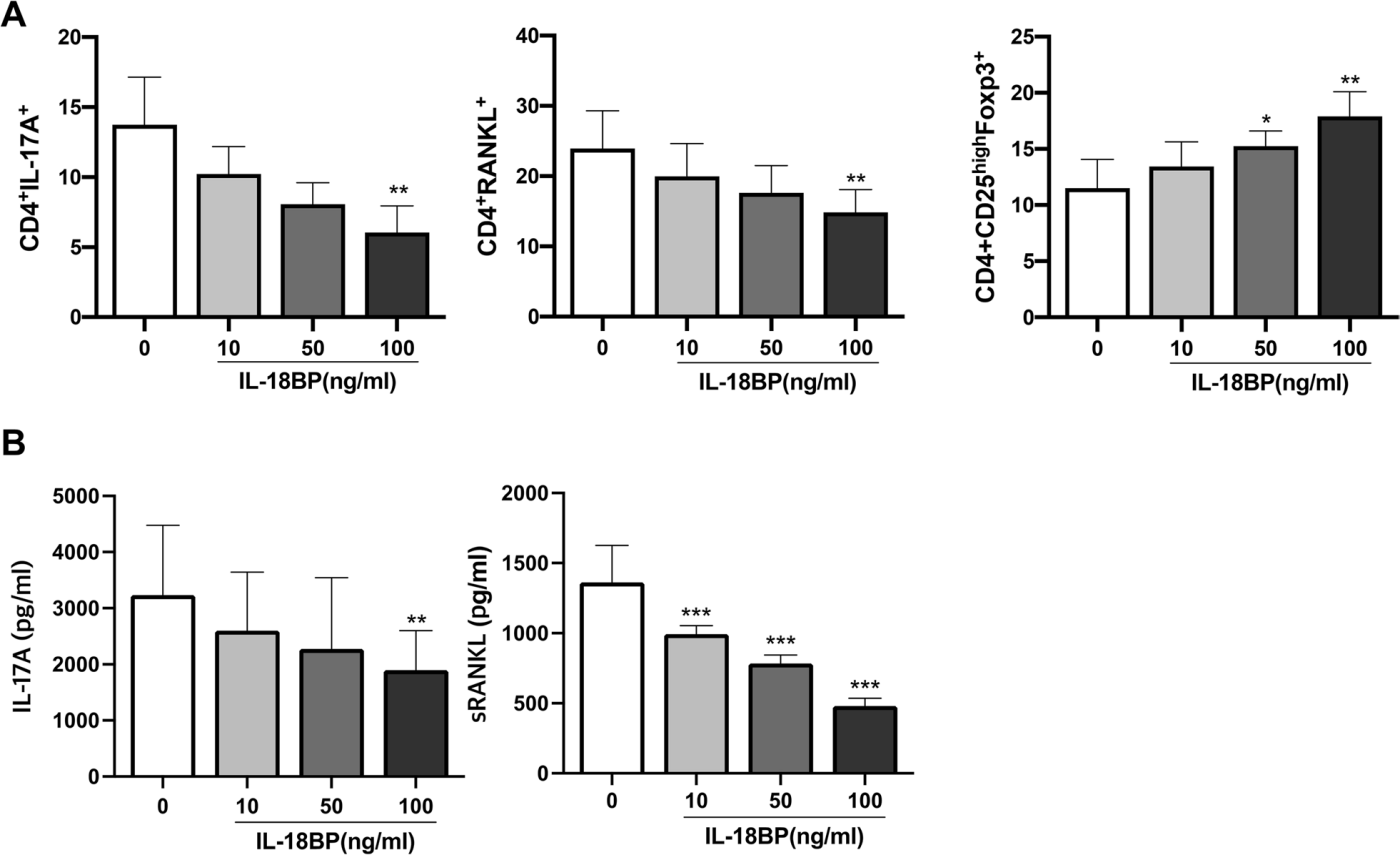
CD4^+^ T cell differentiation of PBMCs determined by flow cytometry, and levels of IL-17A and soluble RANKL in culture soup under Th17 polarizing condition. PBMCs from RA paitents (N = 5) was cultured under Th17 condition (IL-1β, IL-6, IL-23 added media) with various concentration of IL-18BP (10, 50, 100 ng/mL). (A) CD4^+^ T cell differentiation was measured by flow cytometry. (B) Levels of IL-17A and soluble RANKL in culture soup was determined by ELISA. ^*^P < 0.05, ^**^P < 0.001, ^***^P < 0.001

**Fig. 3:**
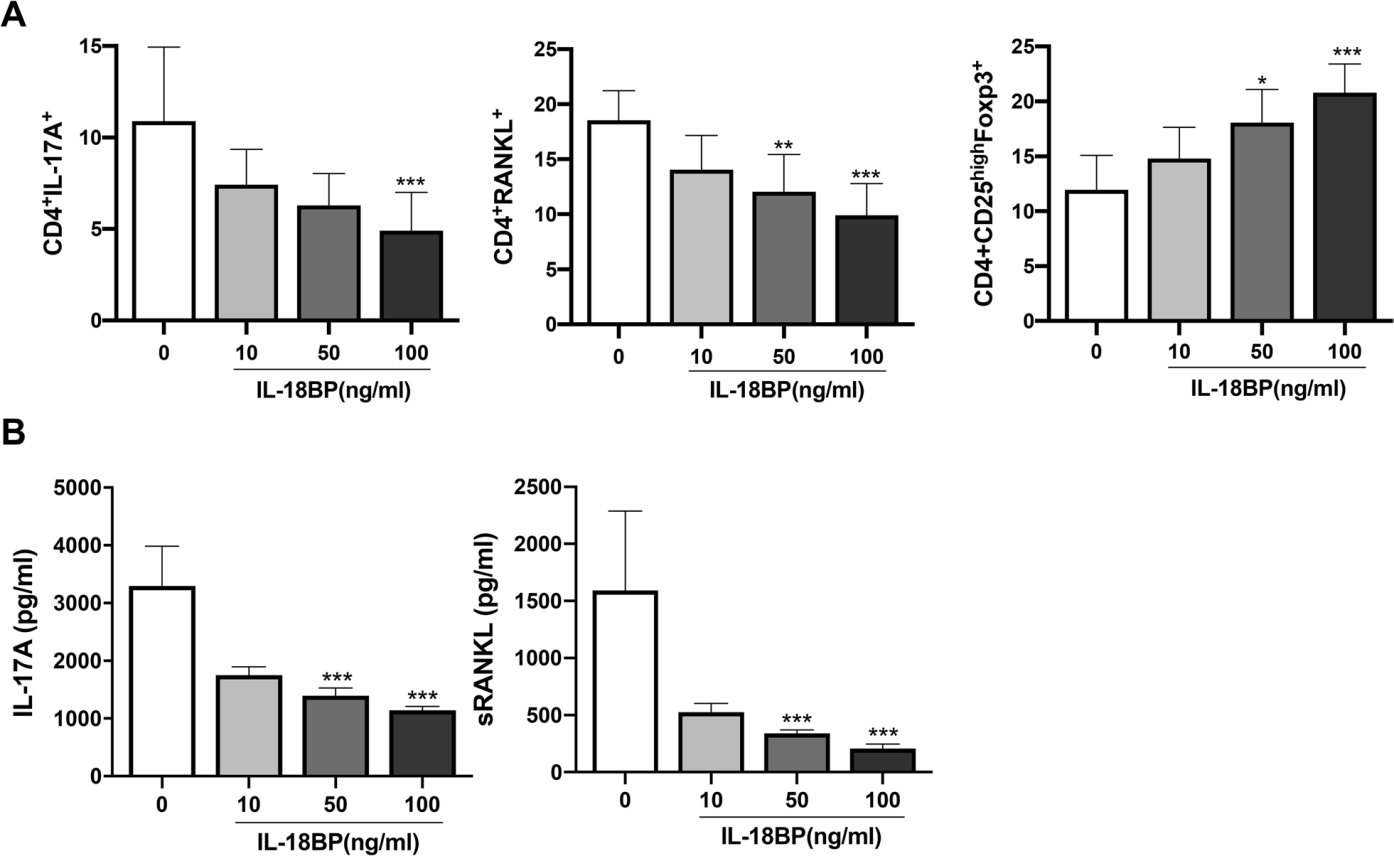
CD4^+^ T cell differentiation of SFMCs (under Th17 polarizing condition) was determined by flow cytometry and levels of IL-17A and soluble RANKL in culture supernate was measured by ELISA. SFMCs from RA patients (N = 20) was cultured under Th17 condition (IL-1β, IL-6, IL-23 added media) with various concentration of IL-18BP (10, 50, 100 ng/mL). **A** CD4^+^ T cell differentiation was measured by flow cytometry. **B** Levels of IL-17A and soluble RANKL in culture soup was determined by ELISA. *P < 0.05, **P < 0.001, ***P < 0.001

To mimic RA condition, T cells were cultured in vitro under Th17 polarising conditions. Flow cytometry analysis of PBMCs showed that IL-18BP dose-dependently decreased the number of CD4^+^ IL-17A^+^ T cells (Th17) and CD4^+^ RANKL^+^ T cells, whereas the number of CD4^+^ CD25^high^ FOXP3^+^ T cells (Treg) was increased (Fig. 2a). Representative flow cytometry images of PBMCs were presented in Figure S1 (Additional file 1). ELISA for IL-17A and soluble RANKL in the PBMC culture medium showed a dose-dependent decrease in IL-17A and sRANKL levels (Fig. 2b). The decrease in CD4^+^ IL-17A^+^ T cell and CD4^+^ RANKL^+^ T cells as well as an increase in CD4^+^ CD25^high^ FOXP3^+^ T cells were also demonstrated in cultured SFMCs (Fig. 3a). Flow cytometry gating strategy of SFMCs were demontrated in Figure S2 (Additional file 2). Similarly, IL-17A and sRANKL levels in the SFMC culture medium decreased proportionally to the IL-18BP dose (Fig. 3b).

### Osteoclast differentiation suppression by IL-18BP under IL-17-induced osteoclastogenesis

**Fig. 4:**
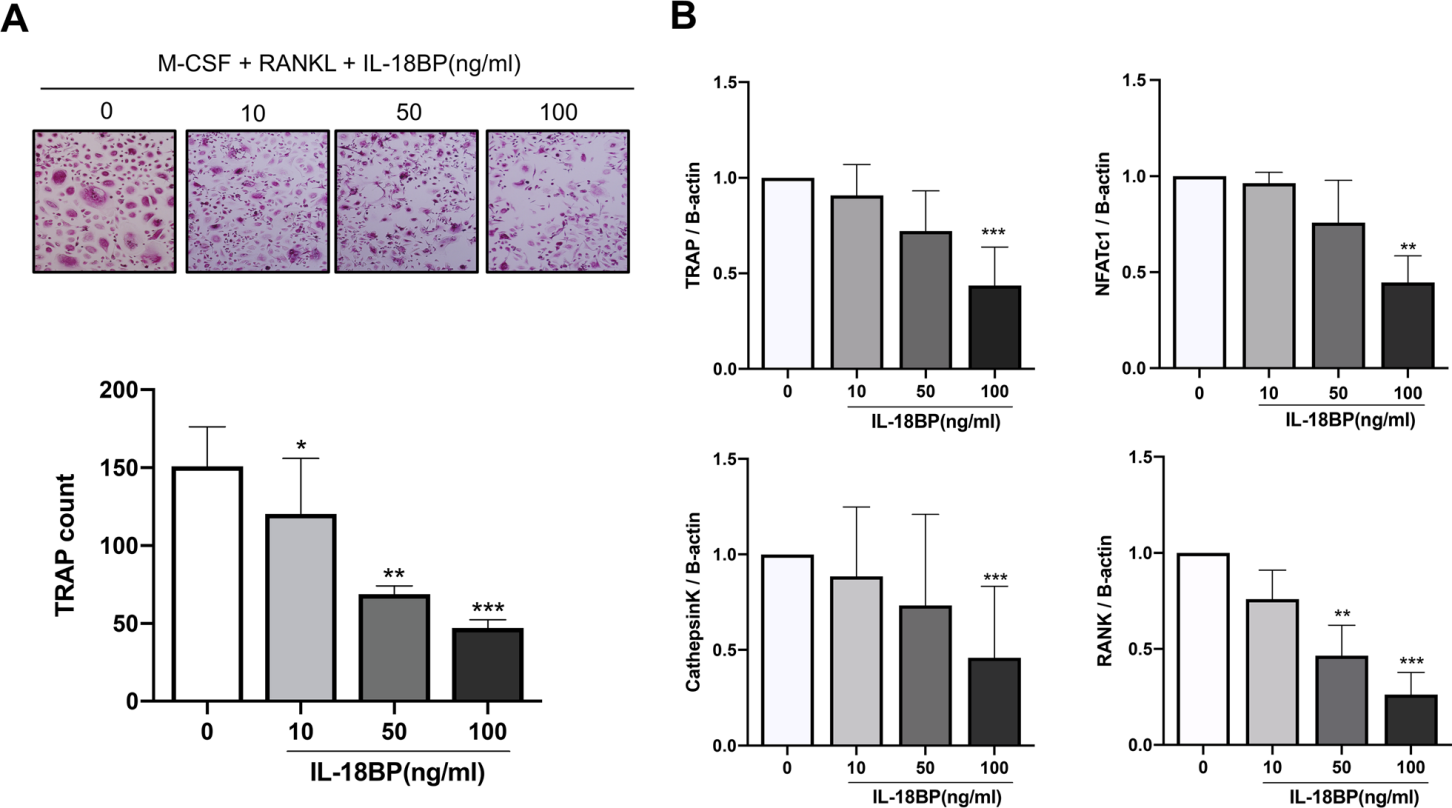
Suppressive role of IL-18BP on osteoclastogenesis under RANKL stimulation. CD14^+^ monocytes from RA patients (N = 5) were cultured with RANKL (30 ng/mL) + M-CSF (25 ng/mL) without or with IL-18BP (10, 50, 100 ng/mL). **A** TRAP^+^ multinucleated cell count was measured. **B** The gene expression of *TRAP, NFATc1, cathepsin K*, and *RANK* from differentiated osteoclasts was measured by real-time quantitative PCR. Data were normalized to beta-actin and reported in relative expression units. ^*^P < 0.05, ^**^P < 0.01, ^***^P < 0.001

**Fig. 5:**
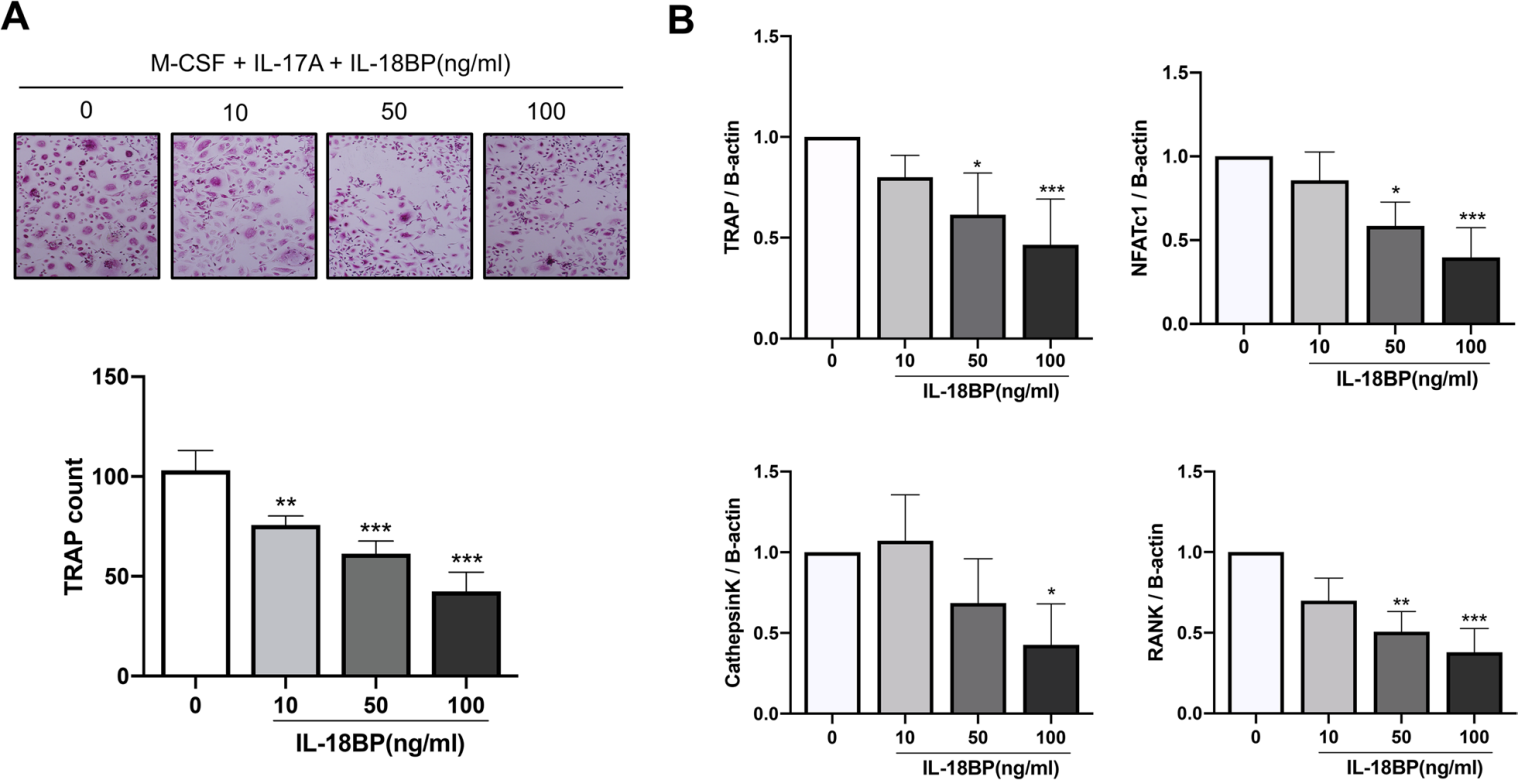
Suppressive role of IL-18BP on osteoclastogenesis under IL-17A stimulation. CD14^+^ monocytes from RA patients (N = 5) were cultured with IL-17A (50 ng/mL) + M-CSF (25 ng/mL) without or with IL-18BP (10, 50, 100 ng/mL). **A** TRAP^+^ multinucleated cell count was measured. **B** The gene expression of *TRAP, NFATc1, cathepsin K*, and *RANK* from differentiated osteoclasts was measured by real-time quantitative PCR. Data were normalized to beta-actin and reported in relative expression units. *P < 0.05, **P < 0.01, ***P < 0.001

CD14^+^ monocytes extracted from human PBMCs were used to determine the effect of IL-18BP on osteoclastogenesis. Increased osteoclast differentiation in inflammatory diseases such as RA is partly mediated by osteoclast-inducing pro-inflammatory cytokines, such as IL-17 [7]. The number of TRAP^+^ cells was dose- dependently decreased by IL-18BP upon the stimulation with RANKL + M-CSF (Fig. 4a). In addition, the expression levels of the osteoclast differentiation-related genes *TRAP, NFATC1*, *CTSK,* and *TNFRSF11A* (*RANK)* decreased in the cells treated with IL-18BP (Fig. 4b). Similarly, treatment with IL-18BP dose dependently decreased the number of TRAP^+^ cells upon the stimulation with IL-17A + M-CSF (Fig. 5a). The expression levels of the osteoclast-related genes was also decreased in the cells treated with IL-18BP, even on the background of stimulation with IL-17A (Fig. 5b).

## Discussion

In the present study, we presented in vitro evidence of the potential of IL-18BP in Th17/Treg regulation and suppression of osteoclastogenesis in RA and revealed the mechanisms underlying its beneficial effects. In the serum and SF of patients with active RA, IL-18BP level was decreased, and serum levels of IL-18BP correlated with the serum level of IL-18 in active RA patients. The Th17/Treg imbalance, which is a pathological hallmark of RA pathogenesis, was counteracted by IL-18BP administration in a dose-dependent manner in vitro. In addition, osteoclast differentiation induced by IL-17A and RANKL in vitro was attenuated by IL-18BP. In addition, RANKL-presenting CD4^+^ T cells were downregulated by IL-18BP in vitro.

Increased Th17 population with decreased Treg population is one of the pathological hallmarks of RA [2], and this Th17/Treg imbalance is one of the treatment targets [22]. The Th17/Treg ratio decreased in patients with RA after tumour necrosis factor α inhibitor treatment [23]. The anti-arthritis effects of other candidates for RA treatment, were also accompanied by changes in the Th17/Treg ratio [24, 25]. In an osteoporosis mouse model, IL-18BP administration recovered bone density with a decrease in the Th17/Treg ratio and pro-inflammatory cytokines [18]. In the present study, Th17 downregulation and Treg upregulation by IL-18BP in vitro Th17 polarising conditions were demonstrated. These were present both in serum and SF of RA patients. Cytokines such as IL-1β, IL-6, and IL-23 are known to induce Th17 differentiation [26], and the addition of the aforementioned cytokines to the culture medium creates Th17 polarizing conditions in vitro [19], mimicking RA situation. The regulation of the Th17/Treg ratio by IL-18BP revealed in the present study is important because it was demonstrated in Th17 polarising conditions (RA mimicking in vitro condition).

Osteoclasts are the main effector cells mediating joint destruction during RA pathogenesis [1]. In one study, IL-18BP was decreased in female patients with osteoporosis, and IL-18BP treatment showed anti-osteoporotic effects in an osteoporosis mouse model [18]. Osteoclastogenesis is increased in inflammatory diseases such as RA, because these conditions are accompanied by increased levels of pro-inflammatory cytokines that have osteoclast-inducing potential [7]. A previous study demonstrated that IL-17, which is increased in patients with RA, augments osteoclastogenesis even in the absence of RANKL [11]. In the present study, IL-18BP suppressed in vitro osteoclastogenesis caused by the stimulation with RANKL and IL-17. Furthermore, IL-18BP administration decreased the number of RANKL-positive helper T cells in vitro. In addition, levels of osteoclast-inducing molecules such as RANKL and IL-17A were lowered by IL-18BP in the culture supernatant of serum and SF PBMCs in vitro.

IL-18 is an inflammatory cytokine, whereas IL-18BP is a naturally occurring protein that blocks the inflammatory response triggered by IL-18 [12]. In the present study, higher levels of IL-18 were found in the serum and SF of patients with active RA, whereas IL-18BP levels were decreased. Furthermore, serum levels of IL-18 and IL-18BP were significantly correlated in active RA, suggesting that IL-18BP may be up-regulated under active inflammatory conditions. Furthermore, we demonstrated that IL-18BP plays a role in immunoregulation (Th17/Treg) and suppression of IL-17-induced osteoclastogenesis. Bresnihan B. et al. demonstrated that levels of IL-18BP did not correlate with joint damage index or inflammatory marker (C-reactive protein). Additionally, IL-18BP was not significantly increased after six months of methotrexate treatment in patients with RA [17]. The previous study compared serum levels of IL-18BP and joint damage/inflammatory marker using a cross-sectional method, and the follow up duration between pre- and post-treatment was short [17], indicating that non-significant correlations between IL-18BP levels and joint damage index may have been observed. Recently, a phase II study of IL-18BP in adult-onset Still’s disease has been conducted, and it showed clinical efficacy via decreasing inflammatory marker and skin lesions [27]. Considering the properties of IL-18BP (naturally occurring antagonist for IL-18), it may potentially be used for RA treatment, alone or in combination therapy with traditional or biologic disease-modifying antirheumatic drugs. The dosage, method of administration, and interval of the previous phase II study of adult-onset Still’s disease [27] may be referred to in further clinical trials of IL-18BP on patients with RA.

Although the present study consistently showed potential beneficial effects of IL-18BP on PBMC and SFMC of RA patients, the effects of IL-18BP were only obtained from the in vitro experiments. This is the most important limitation of the present study. Therefore, further clinical trials should be performed to identify the definite effects of IL-18BP on patients with RA.

## Conclusions

In conclusion, the present study showed that IL-18BP regulated the Th17/Treg cell ratio and suppressed osteoclast formation under Th17 polarizing, RANKL-, or IL-17A- stimulation culture conditions in RA. Further translational studies confirming the results of the present study in vivo could prove the potential therapeutic effects of IL-18BP in RA.

## Supplementary Information

**Additional file 1: Figure S1.** Flow cytometry gating strategy for CD4^+^ RANKL^+^ T cells, CD4^+^ IL-17A^+^ T cells, and CD4^+^ CD25^high^ Foxp3^+^ T cells of RA-PBMCs under Th17 polarizing conditions.

**Additional file 2: Figure S2.** Flow cytometry gating strategy for CD4^+^ RANKL^+^ T cells, CD4^+^ IL-17A^+^ T cells, and CD4^+^ CD25^high^ Foxp3^+^ T cells of RA-SFMCs under Th17 polarizing conditions.

## Data Availability

The datasets generated and/or analyzed in this study are available from the corresponding author upon reasonable request.

## Abbreviations

RA: Rheumatoid arthritis
CD: Cluster of differentiation
IL: Interleukin
Th17: Type 17 helper T cell
Treg: Regulatory T cell
CIA: Collagen-induced arthritis
RANKL: Receptor activator of nuclear factor kappa- Β ligand
IL-18BP: Interleukin-18 binding protein
OA: Osteoarthritis
DAS28: Disease activity score-28 joints
PBMC: Peripheral blood mononuclear cell
Foxp3: Forkhead box P3
SFMC: Synovial fluid mononuclear cell
M-CSF: Macrophage colony-stimulating factor
TRAP: Tartrate-resistant acid phosphatase
RT-qPCR: Real-time quantitative polymerase chain reaction
SEM: Standard error of the mean
