# Supplementary figures and images for: IL-18 binding protein suppresses IL-17-induced osteoclastogenesis and rectifies type 17 helper T cell / regulatory T cell imbalance in rheumatoid arthritis
Source: J Transl Med. 2021 Sep 16;19:392. doi: 10.1186/s12967-021-03071-2 (PMC8444577; doi:10.1186/s12967-021-03071-2)

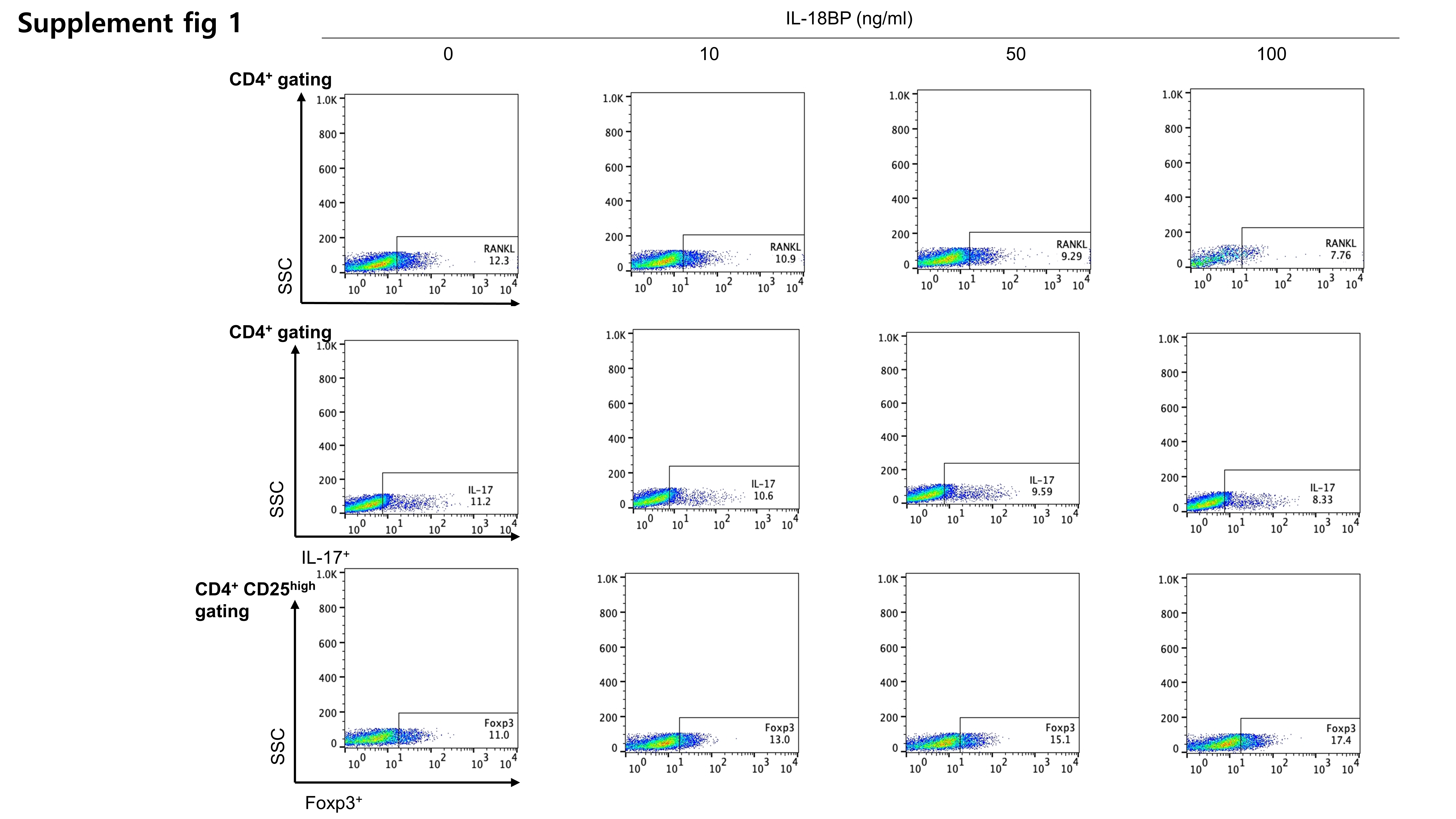

Supplement: Supplementary file 1 — Additional file 1: Figure S1. Flow cytometry gating strategy for CD4+ RANKL+ T cells, CD4+ IL-17A+ T cells, and CD4+ CD25high Foxp3+ T cells of RA-PBMCs under Th17 polarizing conditions. [file 12967_2021_3071_MOESM1_ESM.tif]

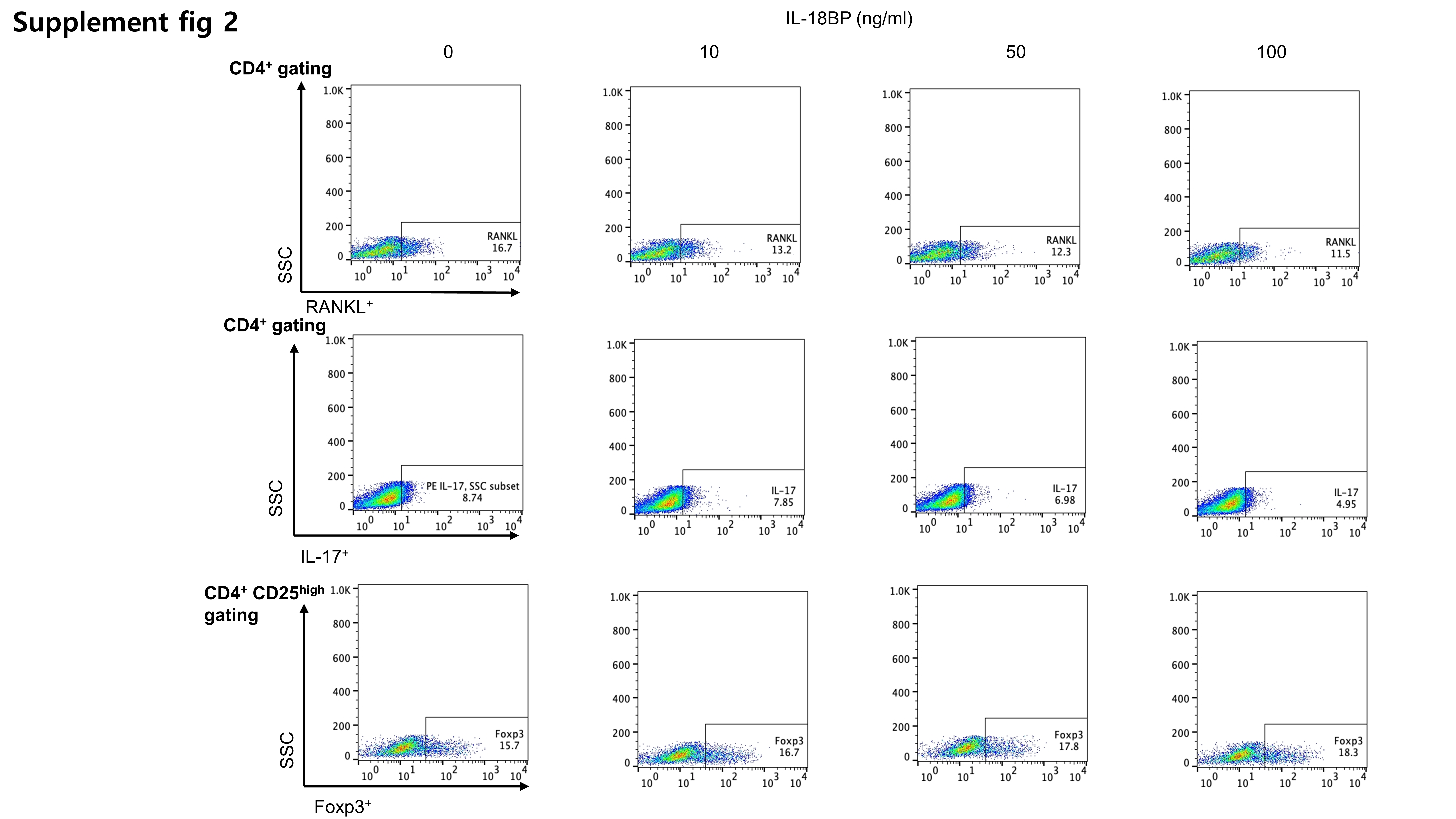

Supplement: Supplementary file 2 — Additional file 2: Figure S2. Flow cytometry gating strategy for CD4+ RANKL+ T cells, CD4+ IL-17A+ T cells, and CD4+ CD25high Foxp3+ T cells of RA-SFMCs under Th17 polarizing conditions. [file 12967_2021_3071_MOESM2_ESM.tif]
